# HIV and lung cancer: a single cancer center experience

**DOI:** 10.3389/fonc.2025.1600709

**Published:** 2025-10-01

**Authors:** Julia Palecki, Matthew Tucker, Andrew Bernstein, Garrett Melby, Tingting Zhan, Ida Micaily

**Affiliations:** ^1^ Department of Internal Medicine, Thomas Jefferson University, Philadelphia, PA, United States; ^2^ Sidney Kimmel Medical College, Thomas Jefferson University, Philadelphia, PA, United States; ^3^ Sidney Kimmel Comprehensive Cancer Center, Department of Medical Oncology, Thomas Jefferson University, Philadelphia, PA, United States

**Keywords:** lung cancer, PLWH, HIV, immunotherapy, antiretroviral (ARV) therapy

## Abstract

Lung cancer (LC) is the leading cause of cancer death in people living with HIV (PLWH) and disproportionately affects this group regardless of CD4 count. At the Sidney Kimmel Comprehensive Cancer Center (SKCCC) at Jefferson Health, we treat an underserved PLWH population with LC and aim to study their clinical course, as they are often excluded from LC trials. We retrospectively reviewed 36 PLWH diagnosed with LC at Jefferson Health from 2016 to 2023. We found that 24 patients were male, 20 patients were Black, and that the median age of LC diagnosis is 66 years (range 38-83 years). 27 patients had non-small cell lung cancer (NSCLC) (20 adenocarcinoma, 7 squamous), 3 had small cell lung cancer (SCLC). 24 patients had undetectable viral loads, and 35 patients were on antiviral therapy. Advanced-stage disease was seen in 32 patients, with a 1.3-year OS (95% CI: 0.8-3.8 years). Of 18 patients with molecular testing available, 4 had KRAS mutations, and 1 had targetable EGFR mutations. Of 16 tested for PD-L1, 8 patients had 1-49% expression, and 1 patient had >50%. Our cohort showed more advanced disease at presentation, younger age at diagnosis, and poor OS despite most patients having undetectable HIV viral loads on antiviral therapy, suggesting a link between well-controlled HIV and aggressive LC that warrants further study.

## 1 Introduction

Lung cancer (LC) has been identified as the leading cause of cancer-related deaths in people living with HIV (PLWH) (1). The elevated risk of lung cancer among patients with HIV, ranging from 2 to 7 times that of the general population, was traditionally thought to be primarily due to the higher smoking prevalence in this population (2–5). There are two main subtypes of lung cancer: NSCLC and SCLC. HIV infection itself is independently associated with an increased risk of non-small cell lung cancer (NSCLC) (6, 7), with two recent meta-analyses revealing that the risk of lung cancer is more than two-fold higher in PLWH, even after accounting for the increased smoking rates within this group (1). Additionally, these patients tend to be diagnosed at a significantly younger age, with the median age of 66 years old in our cohort, compared to 71 years old in the general population (3, 7). A 2007 meta-analysis by Grulich et al., which compared data from multiple studies involving PLWH (n=444,172) and solid organ transplant recipients (n=31,977), showed a similar pattern of increased incidence of 28 different cancer types, including lung cancer. This suggests that the immunomodulatory properties of HIV may be a contributing factor to this heightened cancer risk (8).

The same meta-analysis by Grulich et al. demonstrated that patients with HIV also have notably poorer survival outcomes when diagnosed with lung cancer, even when treatment modality and disease stage are controlled for in the analysis (8). This finding was corroborated by large-scale longitudinal studies conducted on Medicare enrollees from 1996 to 2007, which found that median survival was significantly shorter among HIV-infected NSCLC patients compared to their HIV-negative counterparts. This discrepancy remained even when stage at presentation and access to appropriate lung cancer treatments did not differ between the two groups (9). Other studies, conducted in 2005 and 2015, found the 2-year survival rate for HIV-positive individuals with NSCLC to be approximately 10%, in contrast to 31% for the general population (1, 10). This stark disparity in survival persists even when treatment modality and CD4 count are controlled for, indicating the presence of other, poorly understood factors in HIV-infected patients that may negatively impact their cancer outcomes (11). Several hypotheses have been proposed to explain this phenomenon, including direct viral oncogenesis through Tat expression, chronic lung parenchymal inflammation, or immunosuppression (12). While the relationship between CD4 count and lung cancer risk has not been shown to be significant, the use of highly active antiretroviral therapy (HAART) has been associated with improved survival outcomes in PLWH with NSCLC (13, 14).

HIV also significantly impacts the treatment of lung cancer. A large-scale study published in 2018 found that after adjusting for factors such as age, cancer stage, race, sex, and histologic subtype, only 60.3% of HIV-positive patients were offered treatment, compared to 77.5% of HIV-negative patients. This difference was statistically significant (15). NSCLC comprises a heterogenous group of tumors that vary by molecular expression of various mutations, such as KRAS, EGFR, program death receptor ligand 1 (PD-L1) and others. PD-L1 is in maintaining homeostasis by preventing autoimmunity and regulating T-cell reactivity; in tumor cells, overexpression of PD-L1 leads to immune evasion by cancer cells and inhibition of T cells. In HIV, PD-L1 is overexpressed on viral T-cells and therefore impair immune function (7, 16). Studies have shown no notable difference in PD-L1 expression rates between HIV-positive patients and the general population (1). However, additional studies have observed a higher rate of immune cell infiltration—including CD8+ cytotoxic T-cells, B-cells, and macrophages—into NSCLC tumors among HIV-positive patients when compared to similar tumors in HIV-negative individuals (1). A review published by et al. in JAMA Oncology in 2019 found that pembrolizumab and other PD-L1 inhibitors were safe for use in HIV-positive patients and did not appear to interact with HAART therapy (16). The phase 2 DURVAST trial, which enrolled 20 PLWH with undetectable viral loads and advanced solid tumors (14 of whom had NSCLC), demonstrated that durvalumab administered every four weeks immunotherapy was safe and feasible. The side effect profile was like that of the general population, with no detectable HIV viremia during treatment and a 50% response rate (17). Similar findings regarding safety and efficacy were noted in a study involving seven PLWH with metastatic NSCLC treated with anti-PD-L1 inhibitors such as nivolumab or pembrolizumab (18). Despite these promising results, many larger clinical trials investigating the safety and efficacy of PD-L1 inhibitors and immunotherapy have excluded patients with HIV, though emerging research like these studies suggests that such treatments may be safe and effective for this group.

With the emergence of immunotherapy as the preferred first-line treatment for lung cancer in patients without evidence of a targetable driving mutation, patients with immune dysregulation, such as those living with HIV, pose a multidisciplinary challenge (19, 20). People living with HIV are at an increased risk for lung cancer and experience worse clinical outcomes with fewer treatment options when diagnosed. In the absence of high-powered data to guide treatment decisions for these patients, immunotherapy is often avoided. To address this gap, we identified a subgroup of lung cancer patients living with HIV within the Jefferson Health System in Philadelphia, PA, to investigate their disease characteristics, treatment modalities, and survival outcomes. Our goal is to provide better insight into the appropriate management of these complex patients and inform the development of tailored treatment strategies for PLWH diagnosed with lung cancer.

## 2 Materials and methods

This is a single-center, retrospective study investigating patients living with HIV who were diagnosed with lung cancer at Jefferson Health in Philadelphia, PA, over a period spanning from January 1, 2016, to July 1, 2023.

Following review and approval of the research protocol by our IRB, a retrospective database was created with patients who carried the diagnoses of HIV and lung cancer within our hospital system. Qlik software was used to identify patients (>18 years) within our electronic medical records who carried confirmed diagnoses of both lung cancer and HIV by specific ICD-10 codes after which chart review was performed. Only patients with a confirmed histopathologic diagnosis of lung cancer and a history of documented HIV infection were included.

Multiple clinical variables such as basic demographics, details of immune dysregulation such as underlying diseases and their treatments, as well as cancer-specific information like age at diagnosis, disease stage, histological subtype, molecular profile, and treatment history. Outcomes data, including mortality rates, were recorded and analyzed. This approach allowed for a more detailed understanding of the interplay between immunosuppression, HIV status, and lung cancer outcomes.

Molecular and PD-L1 testing was performed in-house at Thomas Jefferson University Hospital at the time of diagnosis by an internally validated assay. Lung cancer multigene mutational analysis panel by next generation sequencing analysis and RNA fusion panels were performed by an internal test developed and characterized by the Molecular and Genomic Pathology Laboratory of Thomas Jefferson University Hospital. The fusion panel utilizes the Archer FusionPlex Comprehensive Thyroid and Lung Panel, utilizing reverse transcription of RNA from the specimen followed by anchored multiplex PCR to amplify sequences from fusion RNAs. Amplicons are sequenced on an Illumina NextSeq next-generation sequencer. PD-L1 testing was performed utilizing the commercially available Ventana PD-L1 (SP263) assay. This testing was performed on formalin-fixed, paraffin-embedded tumor tissue. No blood ctDNA analysis was used in this study.

The primary outcome of our analysis was to descriptively analyze this patient population and the characteristics of the lung cancer diagnoses utilizing means, standard deviations, medians, and frequencies. Inferential statistics such as t tests and chi square analyses were performed where appropriate. All p-values were two tailed and a level of < 0.05 was considered statistically significant. When appropriate, continuous variables were reported as means ± standard deviations (SD). OS was determined using the Kaplan-Meier method. Analyses were performed using R Studio. Data were anonymized to ensure patient confidentiality, and all analyses were performed in accordance with institutional privacy policies.

## 3 Results

### 3.1 Patient characteristics

**Figure 1:**
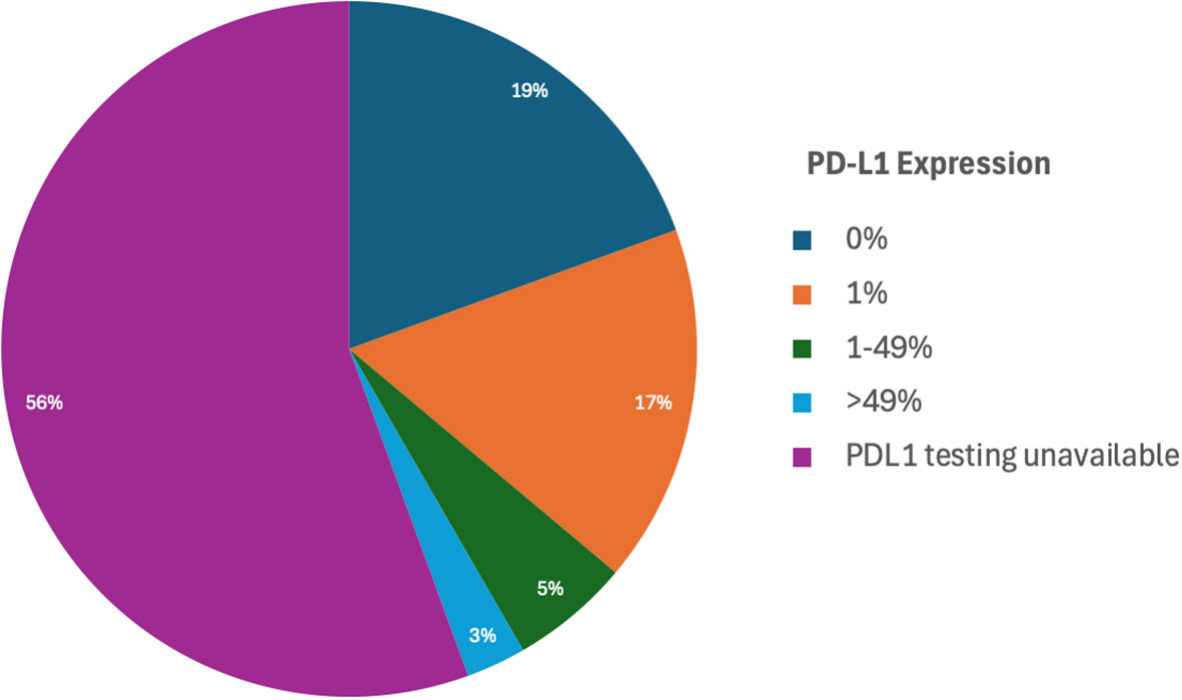
PD-L1 expression rates in LC in PLWH.

This study examined a total of 36 individuals living with HIV (PLWH) who were diagnosed with lung cancer (LC) (**Figure 1**). Within this cohort, 24 individuals (66.7%), were male. Additionally, 20 of the patients (55.6%) identified as Black. The mean age at the time of LC diagnosis for the group was calculated to be 66 years, with a range of 38 to 83 years. Regarding HIV control at the time of their cancer diagnosis, 24 patients (66.7%) were reported to have an undetectable viral load. Furthermore, nearly all the individuals, 35 out of 36 (97.2%), were receiving antiretroviral therapy (ART) at the time of their LC diagnosis. Smoking history was also highly prevalent among the cohort, as 35 (97.2%) of the participants were current or former smokers. On average, their pack-year history was 38.1, with a standard deviation of 18.6 (see **Figure 1** for demographic data).

### 3.2 Lung cancer characteristics

When examining cancer histology in this study, 27 out of 36 patients (75%) were diagnosed with non-small cell lung cancer (NSCLC). Adenocarcinoma was identified as the most common subtype, affecting 20 individuals (55.6%). Squamous cell carcinoma was present in 7 patients (19.4%). In addition to these NSCLC cases, a smaller subset of 3 patients (8.3%) was diagnosed with small cell lung cancer (SCLC), highlighting the variation in lung cancer types among the cohort.

Out of the 36 individuals included in the study, the majority, 27 patients (88.9%), presented with advanced-stage (III, IV) disease at the time of their lung cancer diagnosis. More specifically, 9 individuals (25%) were classified as having stage III lung cancer, while a larger group of 18 patients (50%) had stage IV disease. 9 patients (25%) were diagnosed with early stage I disease, which underscores the late presentation that is common among this population. Interestingly, no patients were diagnosed with stage II disease in this cohort.

Molecular testing was conducted on a subset of 18 patients, providing insights into genetic alterations and biomarkers. Of these, 4 patients (22.2%) were found to harbor KRAS mutations (KRASG12c, KRASG12V and others). These 4 patients had variable PD-L1 levels. Only one patient (5.6%) was identified as having a targetable EGFR mutation and was treated with targeted therapy. Of note, this patient was the only patient with high PD-L1 expression. The remaining patients did not have additional testing performed due to the practice pattern at that time.

Additionally, PD-L1 expression testing was performed in 16 individuals to assess the potential for immunotherapy targeting (**Table 1**). Among the 16 patients with available PD-L1 data, 7 patients (43.8%) exhibited no PD-L1 expression (0%), while 8 patients (50%) demonstrated PD-L1 expression levels in the range of 1-49%. A single patient (6.2%) showed PD-L1 expression greater than 50%, however this patient also had a concurrent targetable EGFR exon 19 deletion and was treated with targeted therapy per standard of care guidelines. These findings provide a detailed understanding of the molecular and histological characteristics within this cohort. Unfortunately, PD-L1 testing was not performed on the remaining patients.

**Table 1:** Characteristics of patients living with HIV and lung cancer.

Characteristic	Value
Median Age, years (range)	66 (38-83)
Sex, (%)	
Male	66.7%
Race, (%)	
African American	55.6%
HAART Use	97.2%
HIV Viral Load, (%)	
Undetectable at time of diagnosis	66.7%
Smoking, (%)	97.2%
Average Pack-Year	38.1
Stage, n (%)	
I	9
II	0
III	9
IV	18
Histology, n (%)	
Adenocarcinoma	20
Squamous cell carcinoma	7
Other	9
PD-L1, n (%)	
0%	7
1%	6
1-49%	2
>49%	1
PD-L1 testing unavailable	20
Treatment, n (%)	
Surgery alone	5 (13.9%)
Radiation alone	8 (22.2%)
Combined therapy	15 (41.7%)
Immunotherapy	4 (11.1%)
No therapy	4 (11.1%)
Overall Survival (years)	1.3

Six patients were treated with immunotherapy-containing regimens. Four of the six patients were treated with immunotherapy (pembrolizumab or nivolumab) alone. One patient was treated with chemoimmunotherapy (carboplatin, pemetrexed, pembrolizumab), and another patient was treated concurrent chemoradiation followed by immunotherapy (durvalumab) maintenance for one year.

### 3.3 Outcomes

The overall survival (OS) for the cohort was determined to be an average of 1.3 years. Six patients underwent immunotherapy as part of their LC treatment regimen. Of these six patients treated with immunotherapy, five individuals (83.3%) died within one year of initiating the treatment. These findings underscore the aggressive nature of the disease in this cohort and suggest the need for further investigation into the effectiveness of immunotherapy and other treatment strategies for this specific population.

## 4 Discussion

There is a notable lack of information on LC in PLWH, as they are often excluded from studies. This study provides a unique glimpse into patient demographics, disease characteristics, genetic data, and treatment data for this underrepresented group and reveals several concerning trends. Patients in this group were diagnosed at a notably young median age, presented with predominantly advanced disease, and experienced significantly poor overall survival rates. This raises concerns regarding the adequacy of current lung cancer screening practices for PLWH and highlights the ongoing challenges related to early detection in this vulnerable population.

The overall survival rates for PLWH diagnosed with lung cancer remain low, which likely reflects both the late-stage diagnoses and the inherent limitations in treatment options available to these patients. This situation is particularly striking when considering that most individuals in our cohort were receiving antiretroviral therapy and had an undetectable HIV viral load at the time of their cancer diagnosis. The persistence of these concerning trends, despite well-managed HIV, raises questions about the potential relationship between effectively controlled HIV and the development of aggressive lung cancer. As our cohort is derived from a single, urban center and our data is retrospective, our findings are hypothesis generating and require larger studies to provide improved generalizability for this population. Data was not able to be easily obtained about the duration of ART, CD4+ T cell counts or viral loads throughout therapy. Our patient population reported in this study incorporates a particularly vulnerable, inner-city, and predominantly Black male population. Additionally, a small sample size without a control group comparator prevents comparator analyses. As noted in larger population studies, PLWH with LC have worse clinical outcomes than cohorts with LC that do not have HIV (8, 9). These limitations should be considered when extrapolating from our findings.

A key limitation of this retrospective cohort report is that while findings are hypothesis-generating, they do not elucidate the mechanisms driving poor outcomes in PLWH with LC. This gap highlights the need for further investigation with both basic and translational research to better characterize the pathogenesis of LC in PLWH, with a focus on the role of chronic inflammation and immune dysregulation. Given that most of our patient cohort had well-controlled HIV and were on ART, we consider other potential factors that may contribute to the intersectionality of their poor outcomes. This can include chronic immune dysregulation in the setting of HIV and potential therapeutic interactions interfering with the pharmacologic properties of ART and systemic cancer-directed therapies. These specific areas can be considered in future translational and pharmacokinetic studies, as it is beyond the scope of our retrospective cohort study. It should be noted that our patient population includes a very high smoking prevalence (97.2%) with a 38.1 pack year (mean), and this is a critical confounding variable in the interpretation of our outcomes. The smoking rate seen in our cohort is similar or higher to the rates seen in similar population studies (8, 9). A future potential clinical study would be utilizing local data on a similar population with high smoking rates and LC that are HIV- to serve as a comparator. Other studies in PLWH and LC have similar patient populations and outcomes (17, 20, 21).

It should be noted that our patient catchment area includes a healthcare disparate population in a large, urban population. We continue to strive to overcome barriers in lung cancer screening in this population and to improve healthcare access in the city of Philadelphia. Further research directions should also include retrospective analyses and multi-institutional studies with diverse populations. This study population consists primary of individuals from an inner-city setting, who often face significant barriers related to social determinants of health, and thus these factors may limit the generalizability of findings to broader populations of PLWH. To advance clinical understanding, pragmatic prospective clinical trials are essential. Currently, there is an open AIDS Malignancy Consortium clinical trial (AMC-111; NCT04949464) investigating LC screening in PLWH, which may provide valuable insight management strategies in this high-risk population.

Furthermore, our data sheds light on the prevalence of oncogenic mutations in lung cancer in PLWH and underscores a critical gap in research investigating immunotherapy for lung cancer in this group. In our study, we found that a significant number of our LC cases in PLWH with molecular testing had targetable mutations, including KRAS, EGFR, and PD-L1. However, unfortunately a larger portion of patients had unknown PD-L1 status in this cohort. Only a minority of PLWH received immunotherapy as part of their lung cancer treatment regimen which may have been due to multiple factors, including the lack of PD-L1 status, availability of immunotherapy at the time of therapy, and the sparse data in the use of immunotherapy in this patient population. Of the six patients who received immunotherapy as part of their therapy, five patients received anti-PD-1 therapy in the front-line setting either alone or in combination with chemotherapy, and one patient received anti-PD-L1 maintenance after concurrent chemoradiation. Among these six patients who did receive immunotherapy alone or in combination with other therapies, the one-year survival rate was quite low, which could have also been related to medical comorbidity and poor performance status. It should also be considered that the utilization of chemotherapy and radiation could have affected therapy outcomes. Only one patient in our patient cohort had a PD-L1 >50%, but this patient did not receive immunotherapy as part of their cancer-directed therapy due to a concurrent finding of EGFR exon 19 deletion mutation. This mutation was targetable and treated with appropriate therapy. This study emphasizes the need for comprehensive molecular analysis and PD-L1 testing in PLWH with LC. In the molecularly-guided, precision medicine era, emphasis should be taken to ensure that comprehensive sequencing are performed for all PLWH with LC through validated assays. A major limitation of this cohort study is that the sample sized precludes an ability to stratify overall survival by disease stage, treatment type or molecular subtype. Given this, larger prospective studies are needed on the use of immunotherapy in PLWH with LC. 

We hypothesize that low rates of immunotherapy utilization despite the presence of targetable mutations is largely due to insufficient data regarding the safety and efficacy of immunotherapy in this subset of patients. PLWH are frequently excluded from clinical trials investigating immunotherapies, despite evidence demonstrating that they have targetable mutations that could benefit from such treatments. The unique nature of HIV, which induces chronic inflammation and immune dysregulation, may alter the safety and effectiveness of immunotherapy treatments, even when HIV is adequately treated. Therefore, it remains unclear whether data from non-HIV patients can be directly extrapolated to the PLWH population. This data highlights the need for additional research to investigate the role of immunotherapy in PLWH with lung cancer and to develop evidence-based treatment guidelines for this high-risk group. 

## 5 Conclusion

In conclusion, this study demonstrates significant gaps in the diagnosis and treatment of lung cancer (LC) in people living with HIV (PLWH). This population presents with a young median age at diagnosis, advanced disease stage at initial presentation, and poor survival outcomes, despite being on antiretroviral therapy. The elevated incidence of advanced-stage LC diagnosis and the low rates of immunotherapy usage within this cohort underscores the limitations in current screening and treatment protocols. The current screening and treatment paradigms may not sufficiently address the specific and unique needs of PLWH, particularly when considering the complexities introduced by HIV-related immune dysregulation. Moreover, the identification of targetable mutations in this patient group further emphasizes the need for further clinical trials to evaluate the safety and efficacy of immunotherapy in PLWH, aiming to develop optimized treatment strategies that are better tailored to serve this high-risk population. Our data suggests that enhancing screening protocols, gaining a deeper understanding of immune dysregulation in PLWH, and conducting dedicated studies on immunotherapy in this group may help to significantly improve care and clinical outcomes for PLWH diagnosed with LC.

## Data Availability

The data analyzed in this study is subject to the following licenses/restrictions: This is internal patient information that is linked to this vulnerable population. Given the small sample size and vulnerable patient population, we do not want to link potential identifiable data. Requests to access these datasets should be directed to ida.micaily@jefferson.edu.
